# Effects of early extubation followed by noninvasive ventilation versus standard extubation on the duration of invasive mechanical ventilation in hypoxemic non-hypercapnic patients: a systematic review and individual patient data meta-analysis of randomized controlled trials

**DOI:** 10.1186/s13054-021-03595-5

**Published:** 2021-06-01

**Authors:** Rosanna Vaschetto, Alessandro Pecere, Gavin D. Perkins, Dipesh Mistry, Gianmaria Cammarota, Federico Longhini, Miguel Ferrer, Renata Pletsch-Assunção, Michele Carron, Francesca Moretto, Haibo Qiu, Francesco Della Corte, Francesco Barone-Adesi, Paolo Navalesi

**Affiliations:** 1 Azienda Ospedaliero Universitaria “Maggiore della Carità”, Anestesia e Terapia Intensiva, Novara, Italy; 2 Dipartimento di Medicina Traslazionale, Università del Piemonte Orientale, via Solaroli 17, 28100 Novara, Italy; 3 Warwick Clinical Trials Unit, Warwick Medical School, Warwick University, Gibbet Hill, Coventry, UK; 4 Anestesia e Rianimazione, Dipartimento di Scienze Mediche e Chirurgiche, Università “Magna Graecia”, Catanzaro, Italy; 5 RIICU, Department of Pneumology, Respiratory Institute, Hospital Clinic of Barcelona, IDIBAPS, CibeRes (CB/06/06/0028), University of Barcelona, Barcelona, Spain; 6 Department of Physiotherapy, Centro Universitário Padre Anchieta, UNIANCHIETA, Jundiaí, SP Brazil; 7 Department of Medicine - DIMED, Section of Anesthesiology and Intensive Care, University of Padua, Padua, Italy; 8 Department of Critical Care Medicine, Zhongda Hospital, School of Medicine, Southeast University, Nanjing, 210009 Jiangsu China

**Keywords:** Noninvasive ventilation, Weaning, Hypoxemic acute respiratory failure

## Abstract

**Background:**

Usefulness of noninvasive ventilation (NIV) in weaning patients with non-hypercapnic hypoxemic acute respiratory failure (hARF) is unclear. The study aims to assess in patients with non-hypercapnic hARF, the efficacy of NIV after early extubation, compared to standard weaning.

**Methods:**

In this individual patient data meta-analysis, we searched EMBASE, Medline and Cochrane Central Register of Controlled Trials to identify potentially eligible randomized controlled trials published from database inception to October 2020. To be eligible, studies had to include patients treated with NIV after early extubation and compared to conventional weaning in adult non-hypercapnic hARF patients. Anonymized individual patient data from eligible studies were provided by study investigators. Using one-step and two-step meta-analysis models we tested the difference in total days spent on invasive ventilation.

**Results:**

We screened 1605 records. Six studies were included in quantitative synthesis. Overall, 459 participants (mean [SD] age, 62 [15] years; 269 [59%] males) recovering from hARF were included in the analysis (233 in the intervention group and 226 controls). Participants receiving NIV had a shorter duration of invasive mechanical ventilation compared to control group (mean difference, − 3.43; 95% CI − 5.17 to − 1.69 days, *p* < 0.001), a shorter duration of total days spent on mechanical ventilation (mean difference, − 2.04; 95% CI − 3.82 to − 0.27 days, *p* = 0.024), a reduced risk of ventilatory associated pneumonia (odds ratio, 0.24; 95% CI 0.08 to 0.71, *p* = 0.014), a reduction of time spent in ICU (time ratio, 0.81; 95% CI 0.68 to 0.96, *p* = 0.015) and in-hospital (time ratio, 0.81; 95% CI 0.69 to 0.95, *p* = 0.010), with no difference in ICU mortality.

**Conclusions:**

Although primary studies are limited, using an individual patient data metanalysis approach, NIV after early extubation appears useful in reducing total days spent on invasive mechanical ventilation.

***Trial registration*:**

The protocol was registered to PROSPERO database on 12/06/2019 and available at PROSPERO website inserting the study code i.e., CRD42019133837.

**Supplementary Information:**

The online version contains supplementary material available at 10.1186/s13054-021-03595-5.

## Introduction

Though a life-saving intervention, invasive mechanical ventilation (i-MV) is prone to side-effects and complications [1, 2]. The process of weaning patient off i-MV should be started promptly to make the time spent on i-MV the shortest possible [3]. Weaning has been recently defined as the time between the first separation attempt and successful extubation that leads to either 7 days of continuous spontaneous breathing or intensive care unit (ICU) discharge, whichever comes first and irrespective of the use of noninvasive ventilation (NIV) in the post extubation period [4].

NIV applied immediately after extubation has been proposed as a measure to prevent post-extubation respiratory failure (i.e., prophylactic NIV in high-risk patients) or as an alternative to i-MV in patients not yet ready to be extubated (i.e., NIV to facilitate weaning) [5, 6].

In patients with acute-on-chronic respiratory failure, particularly those secondary to chronic obstructive pulmonary disease (COPD) exacerbations, compared to standard weaning with the endotracheal tube in place, early extubation followed by immediate NIV application reduces rates of weaning failure and ventilator associated pneumonia, duration of mechanical ventilation, ICU and hospital length of stay (LOS), and improves the rate of survival compared to standard weaning with the endotracheal tube in place [6, 7]. Recent guidelines provide a conditional recommendation in favor of this therapeutic approach in hypercapnic patients with acute-on-chronic respiratory failure. The guideline authors were unable to make recommendation in patients with non-hypercapnic hypoxemic acute respiratory failure (hARF), because of scarcity of available data [6]. After completion of these guidelines, however, two properly powered studies have been published, which included many more patients than previous investigations [8, 9].

Therefore, we designed this systematic review and individual patient data meta-analysis (IPD) to re-assess, in a population of patients recovering from an episode of non-hypercapnic hARF, whether NIV after early extubation would reduce the duration of i-MV (primary endpoint), overall time spent on mechanical ventilation (i-MV + NIV), rate of ventilator associated pneumonia (VAP), time from randomization to ICU and hospital discharges, and time from randomization to ICU death (secondary endpoints), when compared to conventional weaning with the endotracheal tube in place.

## Materials and methods

### Search strategy and selection criteria

This systematic review with meta-analysis was conducted in accordance with the Preferred Reporting Items for a Systematic Review and Meta-analysis of Individual Participant Data.

We considered eligible for inclusion all randomized controlled trials (RCTs) comparing early extubation + NIV with standard weaning with the endotracheal tube in place in adult patients with non-hypercapnic (as defined by PaCO_2_ ≤ 50 mmHg and pH ≥ 7.35) hARF and receiving i-MV for more than 48 h. Patients were excluded in the case of (1) ARF secondary to neurological/ neuromuscular disorders, status asthmaticus, chronic obstructive pulmonary disease (COPD), cardiogenic pulmonary edema; (2) body mass index ≥ 30 kg/m^2^; (3) tracheostomy; (4) obstructive sleep apnoea.

Two authors (FM/AP), independently, searched EMBASE, Pubmed/Medline and Cochrane Central Register of Controlled Trials (CENTRAL) bibliographic databases, without language restriction. Our search encompassed a period from database inception to the 1st October 2020. We supplemented this search by searching review articles and reference lists of trial publications. Collaborators were asked if they knew of any additional RCTs.

Search term combinations are detailed in the Additional file 1.

On search completion and after removal of duplicates, two authors (FM/AP), with the help of a third author (RV) in case of discrepancies, independently assessed for relevance all titles identified by the search strategy. Following title screening, the same independent review procedure was adopted for screening of abstracts and, finally, full texts.

### Data analysis

Data were extracted onto a piloted proforma by two authors (RV/FBA) independently. Extracted data included characteristics of the studies, populations, intervention and comparator, and outcomes. Data were checked for sequence generation, data consistency and completeness and baseline imbalance. IPD were obtained from the authors through a process detailed in the Additional file 2.

RCTs included in quantitative synthesis were evaluated using the Cochrane Risk of Bias assessment tool [10]. The following variables were assessed: sequence generation; allocation concealment; blinding of participants, personnel, and outcome assessors; completeness of outcome data; evidence of selective outcome reporting; and other potential threats to validity. We assessed selectivity of reporting either by comparing study protocols against study reports or by specifically asking study authors whether all prespecified outcomes were reported. Two investigators (FM and AP) independently assessed study quality. Details of the assessment are reported in the Additional files 3 and 4.

Our primary endpoint was to determine whether, in adults receiving i-MV due to non-hypercapnic hARF (population), early extubation followed by immediate NIV application (intervention) compared to standard weaning (comparator), reduces the time spent on i-MV, i.e., days spent on i-MV from randomization to ICU discharge (outcome). Secondary endpoints are summarized in the Additional file 4.

### Statistical analysis

We conducted a meta-analysis with one-step and two-step approach, incorporating all available IPD. Only complete case data were included for all trials in the main analyses. Continuous variables were presented in descriptive analyses as mean ± standard deviation (SD), while categorical and binary variables were presented as frequencies (*n*) and percentages (%), as indicated. Data were analyzed on an intention-to-treat basis. Mixed-effects linear regression models were used to model total days of ventilation and the other continuous outcome variables. Time-to-event outcomes were analyzed through parametric survival models, including random effects considering the cluster effect deriving from different studies. Heterogeneity was assessed within 2-stage models using the *I*^2^ statistic. We also performed a leave-one-out sensitivity analysis, alternatively removing one study at a time, to measure how each study affected the overall estimate and to identify studies that potentially drove the results.

All tests were two-sides and performed at the 5% level of statistical significance. Statistical analyses were done using STATA software version 15 (StataCorp).

The protocol was registered to PROSPERO database on 12/06/2019 and available at PROSPERO website inserting the study code i.e., CRD42019133837.

## Results

**Fig. 1:**
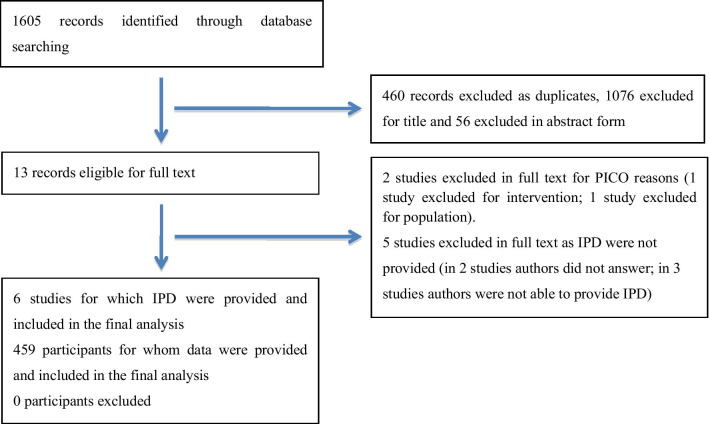
PRISMA-IPD flow diagram. The following flow diagram summarizes the selection process of the randomized control trials included in the systematic review, from the identification to the final phase of data analysis. *IPD* individual patient data, *PICO* patient intervention comparison outcome

Our search identified 1605 records (486 citations in PubMed/Medline, 591 in EMBASE and 528 in the Cochrane Controlled Register of Trials). Following removal of duplicates (*n* = 460), 1076 records were excluded for title and 56 in abstract form. Thirteen full text articles were assessed for eligibility. Seven studies were excluded in full text: 2 for PICO reasons i.e., 1 for intervention and 1 for population and 5 as IPD were not available [11–17]. Six studies were included in the quantitative synthesis [8, 9, 18–21]. Excluded studies and reasons for exclusion are reported in the Additional file 5. The selection process is summarized in the PRISMA-IPD flow diagram (Fig. 1).

Patients meeting all formal inclusion criteria were available for two RCTs [9, 21], while for the remaining 4 studies only selected patients fulfilling inclusion criteria were included (Table 1) [8, 18–20].

**Table 1:** Characteristics of the randomized control trials included in qualitative synthesis

Study	Setting	Primary endpoint	Secondary endpoints	Number of patients included in the original paper	Baseline characteristics of patients at entry into the study	Number of excluded patients and reasons	Number of patients potentially to be analyzed	Number of patients analyzed
Ferrer et al. 2003	2 Spanish hospitals	The decrease of the duration of invasive ventilation defined as positive pressure ventilation delivered through orotracheal intubation or tracheotomy, in the NIV group.	1. Total period of ventilatory support 2. ICU length of stay 3. Hospital length of stay 4. Reintubation 5. Main causes of reintubation - Severe persistent hypoxemia - Severe dyspnoea - Inability to manage secretions - Hemodynamic instability 6. Tracheotomy 7. ICU survival 8. Causes of death within 90d after entry in the study - Septic shock/MOF - Refractory hypoxemia - Cardiac arrest - Pneumothorax - Stroke - Pulmonary embolism	43 patients 21 NIV 22 Control	1. Age 2. Sex 3. Current or former smoker 4. Current of former alcohol abuse 5. APACHE II 6. Duration of ICU stay 7. Duration of mechanical ventilation 8. Number of comorbidities per patient 9. White blood cells 10. Haematocrit 11. Patients with chronic pulmonary disorders 12. Causes of mechanical ventilation - Exacerbation of chronic pulmonary disorders - Congestive heart failure - Community-acquired pneumonia - Hospital-acquired pneumonia - Postoperative respiratory failure - Acute lung injury - Thoracic trauma - Haemoptysis - Cardiac arrest	17 acute-on-chronic exacerbation COPD 9 acute cardiogenic pulmonary oedema 3 severe asthma 8 chronic pulmonary disorder	6 patients 4 Intervention 2 Control	6 patients 4 Intervention 2 Control
Trevisan et al. 2008	Single-centre Brazil	To evaluate the use of bi-level NIV for patients who fail weaning from i-MV	1. ICU length of stay 2. Hospital length of stay 3. otal length of stay in hospital 4. ICU death 5. Ward death 6. Mechanical ventilation time after randomization 7. Total mechanical ventilation time 8. Complications - Pneumonia - Sepsis - Congestive heart failure - Tracheotomy - Return to IMV - Skin necrosis	65 patients 28 NIV 37 Control	1. Age 2. Sex 3. APACHE-II 4. Duration of mechanical ventilation 5. Causes of mechanical ventilation - COPD aggravation and asthma - Heart diseases - Respiratory diseases - Post-surgery respiratory failure - Acute pulmonary lesion - Pneumonia - Tuberculosis - Thoracic trauma	23 acute-on-chronic exacerbation COPD and asthma 11 acute cardiogenic pulmonary oedema 5 PaCO_2_ >50 mmHg and pH >7.35 2 age <18 years old	24 patients 10 Intervention 14 Control	24 patients 10 Intervention 14 Control
Vaschetto et al. 2012	Single-centre Italy	Duration of i-MV	1. ICU length of stay 2. ICU mortality 3. Hospital mortality 4. Extubation failure 5. i-MV before T0 6. i-MV AFTER T0 7. 28-i-MV free days 8. 28-MV free days 9. Weaning 10. Side effects/complications of i-MV Tracheotomy Continuous i.v. sedation	20 patients 10 NIV 10 Control	1. Age 2. Sex 3. APACHE II 4. Causes of mechanical ventilation - Pancreatitis - Pneumonia - Thoracic trauma - Bowel obstruction	None	20 patients 10 Intervention 10 Control	20 patients 10 Intervention 10 Control
Carron et al. 2014	Single-centre Italy	Weaning success/failure rate	1. Duration of i-MV 2. Duration of ventilator support for weaning 3. Duration of total ventilator support 4. Weaning failure 5. Reintubation - Refractory hypoxemia - Bronchial hypersecretion - Transient ischemic attack - Hypercapnia 6. Conventional weaning after reintubation with/without percutaneous dilatational tracheostomy 7. Main complication after entry in the study - VAP - Catheter-related pneumonia - Septic shock - Multiple-organ Failure - Disseminated intravascular coagulation - Cardiogenic shock - Cardiac arrest 8. ICU length of stay 9. Hospital length of stay 10. ICU survival 11. Hospital survival	64 patients 32 NIV 32 Control	1. Age 2. Sex 3. Weight 4. APACHE II 5. ARF hypoxemic hypercapnic (n. of patients) - Exacerbation of chronic pulmonary disease - Asthma - Community-acquired bronchopneumonia - Hospital acquired-bronchopneumonia 6. ARF hypoxemic (n. of patients) - Postoperative respiratory failure - Community-acquired bronchopneumonia - Hospital acquired-bronchopneumonia - Acute cardiogenic pulmonary oedema - Congestive heart failure - Acute pulmonary embolism - Acute pancreatitis - Acute lung injury following ab ingestis - Thoracic trauma - Burn	17 acute-on-chronic exacerbation COPD 1 Asthma 5 acute cardiogenic pulmonary oedema 4 BMI ≥30 10 PaCO_2_ >50 mmHg and Ph >7.35	27 patients 14 Intervention 13 Control	27 patients 14 Intervention 13 Control
Perkins et al. 2018	41 hospitals UK	Time from randomization to successful liberation from all forms of mechanical ventilation	1. Mortality at 30, 90, 180 days 2. Duration of i-MV 3. Duration of total ventilation 4. Time to meeting ICU discharge criteria (defines as no further requirement for level 2/3 care) 5. Reintubation rates 6. Tracheostomy 7. Adverse events and serious adverse events	364 patients 182 NIV 182 Control	1. Age 2. Sex 3. Evidence of delirium 4. Body mass index 5. Duration of ventilation prior to randomization 6. Antibiotics for respiratory 7. Infections 8. APACHE II 9. Admission diagnosis - Pneumonia/respiratory infection - Post-surgery respiratory failure - Cardiac - Non-respiratory infection - Neuromuscular - COPD/asthma exacerbation - Traumatic injuries - GIT bleeding - Pancreatitis - Stroke	15 neuromuscular patients 14 COPD/asthma exacerbation 33 acute cardiogenic pulmonary oedema 48 PaCO_2_ >50 mmHg and pH >7.35	254 patients 130 Intervention 124 Control	254 patients 130 Intervention 124 Control
Vaschetto et al. 2019	6 hospitals China 3 hospitals Italy	1. Days of i-MV - Overall - Medical - Surgical 2. ICU length of stay - Overall - Medical - Surgical	1. Treatment failure 2. Severe events 3. Tracheostomy 4. VAT 5. VAP 6. Use of sedatives 7. Hospital length of stay 8. ICU mortality 9. Hospital mortality	130 patients 65 NIV 65 Control	1. Main causes of i-MV - ARDS - Pneumonia - Septic shock - Polytrauma - Postoperative abdominal surgery - Postoperative vascular surgery - Postoperative thoracic surgery - GIT bleeding - Cerebral bleeding - Pancreatitis 2. Days of i-MV pre-protocol 3. Days of NIV pre-protocol	2 PaCO_2_ >50 mmHg and pH >7.35	128 patients 65 Intervention 63 Control	128 patients 65 Intervention 63 Control

*APACHE II* Acute Physiology and Chronic Health Disease Classification System II, *ARDS* Acute Respiratory Distress Syndrome, *ARF* Acute Respiratory Failure, *BMI* Body Mass Index, *COPD* Chronic Obstructive Pulmonary Disease, *GIT* Gastrointestinal, *ICU* Intensive Care Unit, *i-MV* invasive Mechanical Ventilation, *i.v.* intravenous, *LOS* Length Of Stay, *MOF* Multiple Organ Failure, *N.A.* Not Applicable, *NIV* Non-Invasive Ventilation, *PaCO*_*2*_ arterial partial pressure of carbon dioxide, *PE* Pulmonary Embolism, *UK* United Kingdom, *VAP* Ventilator Associated Pneumonia, *VAT* Ventilator Associated Tracheobronchitis

We conducted the quality assessment only for studies contributing to IPD meta-analysis. All the studies were rated as being at low risk of bias for randomization process, allocation concealment and incomplete outcome data (attrition bias). The inability to blind caregivers to treatment allocation meant that all the studies were at high risk of performance bias. The risk of detection bias was overall low; in 3 studies the strategies to blind outcome assessors from group allocation were described [8, 9, 21], in 2 studies we received description after contacting the authors [18, 20] and for one study the risk remained unclear [19]. One study was not registered in advance [19]. In 2 studies the predefined outcomes were not properly reported [18, 20], encompassing the risk of reporting bias (Additional file 3).

Patient characteristics, stratified by randomization group, are summarized in Table 2. We overall included 459 participants, 233 and 226 in the intervention and control group, respectively, mean (SD) age 62 (15) years, 269 (59%) males. The principal causes for instituting i-MV were post-operative ARF and acute respiratory distress syndrome (ARDS). Surgical and medical patients were 203 (44%) and 256 (56%), respectively. Mean risk of predicted in-hospital mortality based on APACHE [22] or SAPSII [23] scores, varied from 12 to 35% for surgical and medical patients.

**Table 2:** Patient characteristics at ICU admission and ventilator settings and gas exchange at randomization

	Control (n = 226)	Intervention (n = 233)	*p* value
*Characteristics at ICU admission*			
Age, mean (SD)	60 (16)	63 (15)	0.037
Male, n (%)	134 (59%)	135 (58%)	0.769
SAPS II at ICU entry, mean (SD)	44 (15)^*^	45 (17)^†^	0.698
APACHE II at ICU entry, mean (SD)	19 (7)^‡^	19 (7)^§^	0.995
Type of patient (medical vs. surgical)	116/110	140/93	0.059
Main causes of i-MV, n (%)			0.076
ARDS	64 (28%)	57 (25%)	
Pneumonia	19 (8%)	22 (9%)	
Septic Shock	13 (6%)	10 (4%)	
Polytrauma	23 (10%)	28 (12%)	
Post-operative ARF	69 (31%)	51(21%)	
Gastrointestinal Bleeding	7 (3%)	13 (6%)	
Cerebral Bleeding	4 (2%)	2 (1%)	
Pancreatitis	4 (2%)	4 (2%)	
Others	23 (10%)	46 (20%)	
*Ventilator settings and gas exchange at randomization*			
PEEP (cmH_2_O), mean (SD)	7 (2)^°^	7 (2)^°°^	0.451
Pressure Support (cmH_2_O)^§^, mean (SD)	11 (5)^”^	11 (4)^””^	0.414
FiO_2_ (%), mean (SD)	36 (8)	37 (8)	0.124
PaO_2_ (cmH_2_O), mean (SD)	91 (22)^**^	89 (21)^++^	0.287
PaO_2_/FiO_2_ (mmHg), mean (SD)	258 (77)^**^	242 (58)^++^	0.014
pH, mean (SD)	7.43 (0.06)^^^	7.44 (0.05) ^++^	0.748
PaCO_2_ (mmHg), mean (SD)	39 (7)^**^	39 (6)^++^	0.741

*APACHE II* Acute Physiology and Chronic Health Disease Classification System II, *ARDS* Acute Respiratory Distress Syndrome, *ARF* Acute Respiratory Failure, *BMI* Body Mass Index, *FiO*_*2*_ inspired fraction of oxygen, *ICU* Intensive Care Unit, *PaCO*_*2*_ carbon dioxide arterial partial pressure, *PaO*_*2*_ oxygen arterial partial pressure, *PaO*_*2*_*/FiO*_*2*_ oxygen arterial partial pressure and oxygen inspired fraction ratio, *PEEP* Positive End-Expiratory Pressure, *SAPS II* Simplified Acute Physiology Score II, *i-MV* invasive Mechanical Ventilation, *SD* Standard Deviation, *n* number, *vs.* versus

*n = 63, ^†^n = 65, ^‡^n = 154, ^§^n = 162, **n = 225, ^++^n = 232, ^^^n = 226, ^°^n = 224, ^°°^n = 229, ^“^n = 209, ^“”^ n = 217

Criteria for readiness to wean and spontaneous breathing trial before randomization are summarized in the Additional file 6. Ventilator settings and arterial blood gas values at randomization and prior to spontaneous breathing trial (SBT) are also displayed in Table 2. Mean positive end-expiratory pressure (PEEP) and pressure support levels were 7 and 11 cmH_2_O, respectively, in both groups. Noteworthy, PaO_2_/FiO_2_ was slightly though significantly different between intervention 242 (58) mmHg and control group 258 (77) mmHg, (*p* = 0.014).

**Fig. 2:**
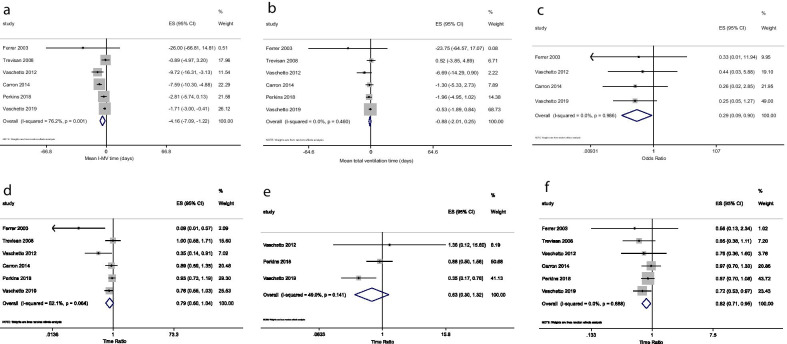
Results of 2-stage IPD-MA. **a** Mean i-MV time (*p* value = 0.006); **b** mean total ventilation time (*p* value = 0.13); **c** occurrence of VAP (*p* value = 0.03); **d** time to ICU discharge (*p* value = 0.09); **e** time to ICU death (*p* value = 0.222), **f** time to hospital discharge (*p* value = 0.009). *CI* confidence interval, *ICU* intensive care unit, *i-MV* invasive mechanical ventilation, *IPD-MA* individual patient data meta-analysis, *VAP* ventilator associated pneumonia

The primary outcome of the study, i.e., length of i-MV, was available for all 459 patients. The two-stage IPD meta-analysis (Fig. 2a) showed a shorter time of i-MV in the treatment group, compared to the control group (mean difference: − 4.16 days; 95% CI − 7.09 to − 1.22; *p* = 0.006). The sensitivity analysis based on the leave-one-out method did not substantially modify the results, which remained statistically significant after exclusion of each study, with point estimates ranging between − 2.70 and − 5.06 days.

The one-stage IPD analysis confirmed the previous results. After adjusting for demographic (age, gender) and severity-related variables (severity scores at ICU admission and PaO_2_/FiO_2_ at randomization), the duration of i-MV remained significantly different between treatment and control group (mean difference: − 3.43 days; 95% CI − 5.17 to − 1.69; *p* < 0.001). Results of random and fixed-effects models did not substantially differ (Table 3).

**Table 3:** Results from 1-stage IPD-MA, according to different models

	Mean i-MV time (days)	Mean total ventilation time (days)	VAP (odds ratio)	Time to ICU discharge (time ratio)	Time to ICU death (time ratio)	Time to hospital discharge (time ratio)
Model 1^*^	− 3.26 (− 5.01 to − 1.50)	− 1.86 (− 3.65 to − 0.06)	0.23 (0.08 to 0.68)	0.82 (0.70 to 0.98)	0.77 (0.49 to 1.22)	0.80 (0.69 to 0.94)
	*p* < 0.001	*p* = 0.042	*p* = 0.008	*p* = 0.027	*p* = 0.273	*p* = 0.006
Model 2^†^	− 3.43 (− 5.19 to − 1.68)	− 2.04 (− 3.84 to − 0.25)	0.25 (0.08 to 0.75)	0.81 (0.69 to 0.96)	0.68 (0.41 to 1.15)	0.81 (0.69 to 0.95)
	*p* < 0.001	*p* = 0.025	*p* = 0.014	*p* = 0.015	*p* = 0.152	*p* = 0.010
Model 3^‡^	− 3.43 (− 5.17 to − 1.69)	− 2.04 (− 3.82 to − 0.27)	0.24 (0.08 to 0.71)	0.81 (0.68 to 0.96)	0.75 (0.45 to 1.23)	0.81 (0.69 to 0.95)
	*p* < 0.001	*p* = 0.024	*p* = 0.014	*p* = 0.015	*p* = 0.251	*p* = 0.011

*Model 1: adjusted by study (fixed-effect model)

^†^Model 2: adjusted by study, age, gender, PaO_2_/FiO_2_, risk score (fixed-effect model)

^‡^Model 3: adjusted by study, age, gender, PaO_2_/FiO_2_, risk score (random effect model)

*ICU* Intensive Care Unit, *IPD-MA* Individual Patient Data Meta-Analysis, *i-MV* invasive Mechanical Ventilation, *PaO*_*2*_*/FiO*_*2*_ arterial partial pressure of oxygen and oxygen inspired fraction ratio, *VAP* Ventilator Associated Pneumonia

Results of two-stage IPD meta-analysis for each secondary outcome are reported in Fig. 2 (panels b–f). The overall duration of mechanical ventilation was similar between intervention group and controls (mean difference: − 0.88 days; 95% CI − 2.01 to 0.25; *p* = 0.130). Time to ICU discharge (time ratio: 0.79; 95% CI 0.60 to 1.04; *p* = 0.09) and mortality (time ratio of 0.63; 95% CI 0.30 to 1.32; *p* = 0.222) were also not significantly different between groups, while the time to hospital discharge (time ratio: 0.82; 95% CI 0.71 to 0.95; *p* = 0.009) and the risk for VAP, (odds ratio: 0.29; 95% CI 0.09 to 0.90; *p* = 0.03) were reduced in the intervention group, as opposed to controls.

Analyses of secondary outcomes based on one-stage approach are reported in Table 3. After adjusting for demographic (age, gender) and severity-related variables (ratio between partial pressure of oxygen and inspired oxygen fraction at randomization and severity scores at ICU admission), all the results became significantly different, except for time to ICU mortality.

As further analysis, reintubation occurrence resulted similar in the two groups as reported in the Additional file 7.

## Discussion

The present IPD meta-analysis shows that, in selected patients recovering from an episode of hypoxemic ARF, early extubation followed by immediate NIV application reduces the duration of i-MV, as opposed to conventional weaning and extubation. Furthermore, compared to standard weaning, early extubation + NIV decreases overall duration of mechanical ventilation, risk of VAP, and time to ICU and hospital discharge.

The study did not identify a significant difference in ICU mortality between the two groups. One possible explanation is that ICU deaths are a relatively rare events (40 cases), leading to an underpowered analysis.

To the best of our knowledge, this is the first IPD meta-analysis of trials investigating the role of NIV in the weaning process of patients recovering from an episode of non-hypercapnic hARF. Previous systematic reviews and meta-analyses addressing the potential of NIV to facilitate weaning [7, 24], considered data on both COPD patients and mixed populations, in the present IPD meta-analysis we analyzed data from 459 patients with non-hypercapnic hARF only, allowing the study to focus on this specific population. By excluding not only hypercapnic patients with COPD or other chronic respiratory disorders, such as neuromuscular disease and obesity-hypoventilation, and those with cardiogenic pulmonary edema, we removed the group of patients who usually show a fast response to NIV.

A recent guideline considers the potential usefulness of NIV in the process of facilitating weaning from i-MV [6]. No recommendation was made for patients with non-hypercapnic hARF due to the paucity of available data. After these guidelines were completed, however, two properly powered studies were published. Both included many more patients than all previous investigations. The first assessed 364 mixed patients, mainly those with non-hypercapnic hARF [8] from 41 ICUs of the UK National Health Service, while the second, 130 non-hypercapnic hypoxemic patients from 9 ICUs, 6 in the Chinese Republic and 3 in Italy [9]. Notably, the results on the time to liberation from i-MV and from any ventilation were largely similar in both cases, showing a shorter duration of i-MV and a similar duration of overall mechanical ventilation, i.e., invasive plus noninvasive. We choose to consider i-MV, rather than the overall duration of mechanical ventilation, as primary endpoint since it has been repeatedly shown to be associated with greater requirement of sedatives, rate of VAP and mortality [7, 25].

Before drawing conclusions, some strengths and limitations of our study require discussion. The major strength is the study design; an IPD meta-analysis is considered to achieve the highest level of evidence and offers several advantages over aggregate patient data meta-analysis [26]. Furthermore, the present work considers only RCTs. If on the one hand our choice excludes observational studies of potential interest, on the other hand it incorporates the studies providing the highest level of evidence. Finally, the amount of missing data was small, and only present for outcomes considered secondary endpoints, in a range from 0 to 3%.

Our meta-analysis has several additional potential limitations. First, we could not include patients from 5 of the identified studies (one of which was available only in abstract form [11]) as in 2 cases we could not reach the authors, while in the other 3 cases datasets were not available [11, 13,14, 16, 17]. Second, the study protocols of the included studies were not identical, as NIV after early extubation was applied before readiness for SBT in two studies [9, 21], after failing one SBT [8, 18, 20] in three RCTs, or after failing SBT for three consecutive days [19] in one study. Nevertheless, the sensitivity analysis based on the leave-one-out method indicates no effect on the primary endpoint. Third, despite the overall risk of bias being assessed as low, blinding the caregivers to treatment allocation was not possible in all the original studies. This is partly mitigated by our choice of objective outcomes, where the risk of detection bias is low. We share this limitation with previous meta-analyses on the use of NIV to facilitate weaning [7, 24]; however, the reporting bias affects IPD meta-analysis to a lesser extent than traditional meta-analysis. Fourth, most of the included studies are of limited size. As a result, baseline imbalances between treatment groups, such as PaO_2_/FiO_2_ values that was different in the intervention and control group could have occurred by chance. However, the results are not substantially affected when adjusting for possible confounders.

## Conclusions

Patients recovering from an episode of hARF may benefit from a weaning strategy based on early extubation followed by immediate NIV application. Compared to conventional weaning, replacing the endotracheal tube with a noninvasive interface reduces the duration of i-MV. Overall time spent on mechanical ventilation, length of ICU and hospital stay, and risk of VAP may also be reduced by this weaning strategy. Future studies are warranted to evaluate whether this approach is also associated with reduced mortality.

## Supplementary Information

**Additional file 1.** Search strategies

**Additional file 2**. Letters sent to the authors

**Additional file 3**. Risk of bias assessment within studies included in quantitative synthesis

**Additional file 4**. Extended methods: secondary outcomes, search strategy, data collection process, and risk of bias (quality) assessment

**Additional file 5**. 1076 studies were excluded considering the title, while 58 after reading the abstract or full text. The table summarize the reason for exclusion of the 58 papers

**Additional file 6**. Criteria for readiness to wean and spontaneous breathing trails performed before randomization

**Additional file 7**. Results of two-stage IPD-MA. Occurrence of reintubation (p value=0.83)

## Data Availability

The datasets used and analysed during the current study are available from the corresponding author on reasonable request.

## Abbreviations

APACHE II: Acute Physiology and Chronic Health Disease Classification System II
ARDS: Acute Respiratory Distress Syndrome
ARF: Acute Respiratory Failure
BMI: Body Mass Index
COPD: Chronic Obstructive Pulmonary Disease
GIT: Gastrointestinal
hARF: Hypoxemic Acute Respiratory Failure
ICU: Intensive Care Unit
i-MV: Invasive Mechanical Ventilation
IPD-MA: Individual Patient Data Meta-Analysis
i.v.: Intravenous
LOS: Length Of Stay
MOF: Multiple Organ Failure
n: Number
N.A.: Not Applicable
NIV: Non-Invasive Ventilation
PaCO_2_: Arterial partial pressure of carbon dioxide
PE: Pulmonary Embolism
UK: United Kingdom
RCT: Randomized Controlled Trials
SAPS II: Simplified Acute Physiology Score II
SD: Standard Deviation
VAP: Ventilator Associated Pneumonia
VAT: Ventilator Associated Tracheobronchitis
vs.: Versus
