# Supplementary material for: Effects of early extubation followed by noninvasive ventilation versus standard extubation on the duration of invasive mechanical ventilation in hypoxemic non-hypercapnic patients: a systematic review and individual patient data meta-analysis of randomized controlled trials
Source: Crit Care. 2021 Jun 1;25:189. doi: 10.1186/s13054-021-03595-5 (PMC8169383; doi:10.1186/s13054-021-03595-5)
Supplement: Supplementary file 1 — Additional file 1. Search strategies [file 13054_2021_3595_MOESM1_ESM.pdf]

Additional file 1.

Search strategy in EMBASE

#1 noninvasive AND ventilation OR (non AND invasive AND ventilation) OR niv OR ventilation

#2 weaning OR (ventilation AND weaning) OR (ventilator AND weaning)

#3 extubation OR (airway AND extubation)

#4 randomized AND controlled AND trial OR (randomised AND controlled AND trial) OR rct OR (controlled AND trial)

#5 #1 AND #2 AND #3 AND #4

Search strategy in the Cochrane Central Register of Controlled Trials (CENTRAL)

#1 "noninvasive ventilation") OR ("non-invasive ventilation") OR (NIV) OR (ventilation)

#2 (weaning) OR (ventilation weaning) OR (ventilator weaning)

#3 (extubation) OR (airway extubation)

#4 (randomised controlled trial) OR (randomized controlled trial) OR (RCT) OR (controlled trial)

#5 #1 AND #2 AND #3 AND #4

Search strategy PubMed

((((((((((positive pressure respiration) OR positive-pressure respiration)) OR (((respiration, artificial) OR respiration artificial) OR artificial respiration)) OR ((mechanical ventilation) OR mechanic ventilation)) OR (((non invasive ventilation) OR non-invasive ventilation) OR NIV)) OR (((intubation, intratracheal) OR intubation intratracheal)) OR intratrachal intubation))) AND ((((ventilation weaning) OR ventilation weaning)) OR (airway extubation) OR extubation))) AND (((((noninvasive ventilation) OR non-invasive ventilatioln) OR NIV)) OR (((noninvasive positive pressure ventilation) OR non-invasive positive pressure ventilation) OR NIPPV)))) AND ((((randomized controlled trial) OR randomised controlled trial) OR RCT) OR trial)
