# Supplementary material for: Effects of early extubation followed by noninvasive ventilation versus standard extubation on the duration of invasive mechanical ventilation in hypoxemic non-hypercapnic patients: a systematic review and individual patient data meta-analysis of randomized controlled trials
Source: Crit Care. 2021 Jun 1;25:189. doi: 10.1186/s13054-021-03595-5 (PMC8169383; doi:10.1186/s13054-021-03595-5)
Supplement: Supplementary file 3 — Additional file 3. Risk of bias assessment within studies included in quantitative synthesis [file 13054_2021_3595_MOESM3_ESM.pdf]

Additional file 3. Risk of bias assessment within studies included in quantitative synthesis.

Author	Selection bias (randomization)	Selection bias (allocation concealment)	Performance bias	Detection bias	Attrition bias	Reporting bias	Other bias
Ferrer et al. 2003	+	+	-	?	+	?	?
Trevisan et al. 2008	+	+	-	+	+	-	+
Vaschetto et al. 2012	+	+	-	+	+	+	-
Carron et al. 2014	+	+	-	+	+	-	+
Perkins et al. 2018	+	+	-	+	+	+	+
Vaschetto et al. 2018	+	+	-	+	+	+	+

**Legend**	
Low risk of bias	+
Unclear	?
High risk of bias	-
