# Supplementary material for: Effects of early extubation followed by noninvasive ventilation versus standard extubation on the duration of invasive mechanical ventilation in hypoxemic non-hypercapnic patients: a systematic review and individual patient data meta-analysis of randomized controlled trials
Source: Crit Care. 2021 Jun 1;25:189. doi: 10.1186/s13054-021-03595-5 (PMC8169383; doi:10.1186/s13054-021-03595-5)
Supplement: Supplementary file 4 — Additional file 4. Extended methods: secondary outcomes, search strategy, data collection process, and risk of bias (quality) assessment [file 13054_2021_3595_MOESM4_ESM.pdf]

Additional file 4.

Title: Effects of Early Extubation Followed by Noninvasive Ventilation versus Standard Extubation on the Duration of Invasive Mechanical Ventilation in Hypoxemic Non-Hypercapnic Patients: A Systematic Review and Individual Patient Data Meta-Analysis of Randomized Controlled Trials.

Rosanna Vaschetto[1,2], MD, PhD; Alessandro Pecere[2*], MSc; Gavin D. Perkins[3], MD; Dipesh Mistry[3], PhD, Gianmaria Cammarota[1], MD, PhD; Federico Longhini[4], MD; PhD; Ferrer Miguel[5], MD; Renata Pletsch-Assunção[6], MD; Carron Michele[7], MD; Francesca Moretto[2], MD; Haibo Qiu[8], MD, PhD; Francesco Della Corte[1,2], MD; Francesco Barone-Adesi[2§], MD, PhD; Paolo Navalesi[7], MD, FERS.

Extended Methods

Secondary outcomes

Secondary objectives are 1) overall duration of mechanical ventilation, i.e., days spent on i-MV (invasive mechanical ventilation) and NIV (noninvasive mechanical ventilation) from randomization to intensive care unit (ICU) discharge; 2) ventilator-associated pneumonia (VAP) i.e., number of patients developing VAP; 3) time o ICU discharge, i.e., days from randomization to ICU discharge; 4) time to ICU mortality, i.e., days from randomization to ICU death; 5) time to hospital discharge, i.e., days from randomization to hospital discharge.
We extracted and analyzed data on 1) overall duration of mechanical ventilation from all 459 patients; 2) time to ICU discharge from 458 patients (233 intervention and 225 control); 3) time to ICU death from 458 patients (233 intervention and 225 control); 4) time to hospital discharge from 439 patients (223 intervention and 216 control); and 5) occurrence of VAP from 205 patients (103 intervention and 102 control). Data on VAP were available from 5 studies only.

Search strategy

Search term combinations consisted of specific words in all fields, text words and word variations for the concepts of population (ventilation, weaning) and intervention/comparator (non-invasive ventilation, invasive ventilation). Clinical trial register (www.ClinicalTrials.gov and the International Clinical Trials Research Platform) was also searched to identify any unpublished trial, in order to evaluate possible publication bias. To complement the database searches, the bibliographies of all relevant primary articles and reviews were also searched manually to identify any articles missed by the electronic searches.

Data collection process

After selection of relevant articles, the corresponding authors of these trials were contacted via email or personal conversation, detailing the objectives of the collaborative meta-analysis, background information and the datasheet for input of individual patient results. If authors' email addresses were missing or obsolete, we searched for authors' recent publications or publicly available profiles. Cover letter sent to the corresponding authors is provided in Appendix 3. If the contacted author did not respond at our first attempt, we tried three more times, before excluding the study. The corresponding authors were also contacted about unpublished data to possibly enlarge the data pool. The authors were contacted when further information was required about random sequence generation methods, allocation concealment and blinding of outcome assessment. All data were merged into a single database and converted to a STATA format for analysis. Data validation was performed, checking the dataset for data entry mistakes and inconsistency, and the study authors were contacted again for any doubt regarding their dataset and to provide missing data, when necessary.

Risk of bias (quality) assessment

RCTs included in quantitative synthesis were assessed using the Cochrane Risk of Bias assessment tool. Two members of the review team (FM/AP), not involved in any included study, independently assessed the risk of bias in each trial within for random sequence generation (selection bias), allocation concealment (selection bias), blinding of participants and personnel (performance bias), blinding of outcome assessment (detection bias), incomplete outcome data (attrition bias), selective reporting (reporting bias). Disagreements were resolved by discussion with a third author (RV). Where the risk of bias for a domain remains unclear, the corresponding authors of the included studies were asked to provide additional information to resolve the uncertainty.
