# Supplementary material for: Effects of early extubation followed by noninvasive ventilation versus standard extubation on the duration of invasive mechanical ventilation in hypoxemic non-hypercapnic patients: a systematic review and individual patient data meta-analysis of randomized controlled trials
Source: Crit Care. 2021 Jun 1;25:189. doi: 10.1186/s13054-021-03595-5 (PMC8169383; doi:10.1186/s13054-021-03595-5)
Supplement: Supplementary file 5 — Additional file 5. 1076 studies were excluded considering the title, while 58 after reading the abstract or full text. The table summarize the reason for exclusion of the 58 papers [file 13054_2021_3595_MOESM5_ESM.pdf]

Additional file 5. 1076 studies were excluded considering the title, while 58 after reading the abstract or full text. The table summarize the reason for exclusion of the 58 papers.

Article	Reason for exclusion
Weaning from Mechanical Ventilation: What Should Be Done When a Patient's Spontaneous Breathing Trial Fails? Munshi L., Ferguson N.D.	Article commentary
Comparison of two opposite strategies of weaning: High vs. low work of breathing: A multicentre randomised controlled trial. Subirà C. et al.	Compares invasive mechanical ventilation to spontaneous breathing trial as weaning methods
The effect of weaning protocol based on spontaneous breathing trial on the duration of mechanical ventilation and weaning time. Jhamb U., Kishore R.	Pediatric population
Proportional assist ventilation versus pressure support ventilation in weaning ventilation: a pilot randomised controlled trial. Botha J. et al.	Compares two modalities of invasive mechanical ventilation as weaning methods
An Open Label Randomized Controlled Trial to Compare Low Level Pressure Support and T-piece as Strategies for Discontinuation of Mechanical Ventilation in a General Surgical Intensive Care Unit. Chittawatanarat K. et al.	Compares invasive mechanical ventilation to spontaneous breathing trial as weaning methods
Effectiveness of continuous positive pressure ventilation in reducing the length of stay in post cardiac bypass surgery patients. Laiq N. et al.	Compares invasive mechanical ventilation to continuous positive airway pressure as weaning methods
Neurally adjusted ventilatory assist as an alternative to pressure support ventilation in adults: a French multicentre randomized trial. Demoule A. et al.	Compares two modalities of invasive mechanical ventilation as weaning methods
Proportional assist ventilation versus pressure support ventilation as weaning modes for mechanically ventilated patients: A randomised controlled trial. Botha J. et al.	Compares two modalities of invasive mechanical ventilation as weaning methods
A pilot randomized trial comparing weaning from mechanical ventilation on pressure support versus proportional assist ventilation. Bosma K.J. et al.	Compares two modalities of invasive mechanical ventilation as weaning methods
Automatic tube compensation versus pressure support ventilation as a weaning mode, does it make a difference? Wafy S. et al.	Compares invasive mechanical ventilation to spontaneous breathing trial as weaning methods
Discontinuation of ventilatory support: New solutions to old dilemmas. Peñuelas O. et al.	Review
Comparison of neurally adjusted ventilatory assist and pressure support ventilation during the early phase of weaning from mechanical ventilation - A randomised controlled study. Demoule A. et al.	Compares two modalities of invasive mechanical ventilation as weaning methods
Comparison of proportional assist ventilation plus, T-tube ventilation, and pressure support ventilation as spontaneous breathing trials for extubation: A randomized study. Teixeira S.N. et al.	Compares two modalities of invasive mechanical ventilation and spontaneous breathing trial as weaning methods
Noninvasive ventilation in withdrawal from mechanical ventilation. Ferrer M. et al.	Review
Noninvasive positive-pressure ventilation as a weaning strategy for intubated adults with respiratory failure. Burns K.E. et al.	Review

Noninvasive ventilation immediately after extubation improves weaning outcome after acute respiratory failure: A randomized controlled trial. Ornico S.R. et al.	Compares non-invasive ventilation to standard oxygen therapy as weaning methods
Preventive use of noninvasive ventilation after extubation: A prospective, multicenter randomized controlled trial. Su C.-L. et al.	Compares non-invasive ventilation to a standard medical therapy as weaning methods
A five-year series on the use of noninvasive ventilation as a weaning tool from invasive ventilation. Kee A. et al.	Prospective observational study without groups of intervention
Noninvasive ventilation to shorten the duration of mechanical ventilation. Epstein S.K.	Review
Non-invasive ventilation. Nava S. et al.	Review
Benefits of non-invasive ventilation after extubation in the postoperative period of heart surgery. Lopes C.R. et al.	Compares non-invasive ventilation to standard oxygen therapy as weaning methods
Non-invasive ventilation in the weaning process. Ferrer M.	Review
Noninvasive ventilation to prevent respiratory failure after extubation in high-risk patients. Nava S. et al.	Compares non-invasive ventilation to a standard medical therapy as weaning modes in hypercapnic patients
VENISE: Non-invasive ventilation for the weaning of mechanical ventilation in acute-on-chronic respiratory failure patients. A prospective randomised controlled and multicenter trial. Girault C. et al.	Population with acute-on-chronic hypercapnic respiratory failure
Noninvasive positive pressure ventilation as a weaning strategy for intubated adults with respiratory failure. Burns K.E. et al.	Review
Noninvasive ventilation during weaning from mechanical ventilation. Ferrer M. et al.	Review
Ventilator modes used in weaning. Hess D.	Review
Noninvasive ventilation. Mehta S., Hill N.S.	Review
The usefulness of noninvasive positive pressure ventilation as a new weaning method. Tae Sun Shim et al.	Considers only non-invasive ventilation as weaning methods
Noninvasive ventilation as a systematic extubation and weaning technique in acute-on-chronic respiratory failure: A prospective, randomized controlled study. Girault C. et al.	Population with acute-on-chronic hypercapnic respiratory failure
The role of continuous positive airway pressure during weaning from mechanical ventilation in cardiac surgical patients. Bailey C.R. et al.	Considers only continuous positive airway pressure as weaning methods
Randomised controlled trial of weaning by patient triggered ventilation or conventional ventilation. Chan V., Greenough A.	Pediatric population
Targeted-Volume Noninvasive Ventilation Reduces Extubation failure in Postextubated Medical Intensive Care Unit Patients: A Randomized Controlled Trial. Thanthitaweewat V. et al.	Compares non-invasive ventilation to standard oxygen therapy as weaning methods
Does bilevel positive airway pressure improve outcome of acute respiratory failure after open-heart surgery? Elgebaly AS.	Compares invasive mechanical ventilation to non-invasive ventilation but not as weaning methods

Effects of Noninvasive Positive-Pressure Ventilation with Different Interfaces in Patients with Hypoxemia after Surgery for Stanford Type A Aortic Dissection. Yang Y. et al.	Considers only non-invasive ventilation
The role of noninvasive ventilation in the ventilator discontinuation process. Hess D.	Review
Noninvasive mechanical ventilation in patients with acute respiratory failure after cardiac surgery. Kilger E. et al.	Considers only non-invasive ventilation
Noninvasive ventilation during weaning. Laier-Groeneveld G. et al.	Retrospective observational study
The role of noninvasive ventilation for acute respiratory failure. Hamel D.S., Klonin H.	Pediatric population
Effect of early application of biphasic positive airway pressure on the outcome of extubation in ventilator weaning. Jiang J.S. et al.	Compares non-invasive ventilation to standard oxygen therapy as weaning methods
Noninvasive techniques of weaning from mechanical ventilation. Nava S.	Review
Noninvasive positive-pressure ventilation in chronic obstructive pulmonary disease. Nicholson D. et al.	Review
Protocolised trial of invasive and non-invasive weaning off ventilation (The 'Breathe' Study). Perkins G.D.	Study protocol
Randomized control study of sequential non-invasive following short-term invasive mechanical ventilation in the treatment of acute respiratory distress syndrome as a result of existing pulmonary diseases in elderly patients. Du L.L. et al.	Compares invasive mechanical ventilation to continuous positive airway pressure as weaning methods
Noninvasive Positive Pressure Ventilation for Early Extubation of Acute Hypoxemic Respiratory Failure. Nava S.	Clinical trial not completed
Effect of Postextubation High-Flow Nasal Oxygen With Noninvasive Ventilation vs High-Flow Nasal Oxygen Alone on Reintubation Among Patients at High Risk of Extubation Failure: A Randomized Clinical Trial. Thille AW. Et al.	Compares effect of non-invasive ventilation to high-flow nasal oxygen on reintubation rate
High-flow nasal oxygen therapy alone or with non-invasive ventilation in immunocompromised patients admitted to ICU for acute hypoxemic respiratory failure: the randomised multicentre controlled FLORALI-IM protocol. Coudroy R. et al.	Compares effect of non-invasive ventilation to high-flow nasal oxygen on not-intubated patients
Prophylactic Noninvasive Ventilation Versus Conventional Care in Patients After Cardiac Surgery. Liu Q. et al	Metanalysis
Is Protocolised Weaning that Includes Early Extubation Onto Non-Invasive Ventilation More Cost Effective Than Protocolised Weaning Without Non-Invasive Ventilation? Findings from the Breathe Study. Khan I. et al.	Cost-effectiveness analysis
Noninvasive Ventilation as a Weaning Strategy in Subjects with Acute Hypoxemic Respiratory Failure. Shan. M. et al.	Metanalysis
Effect of Sequential Noninvasive Ventilation on Early Extubation After Acute Type A Aortic Dissection. Liu K. et al.	Retrospective study

Neurally Adjusted Ventilatory Assist versus Pressure Support Ventilation in Difficult Weaning: a Randomized Trial. Liu L. et al.	Compares two modalities of invasive mechanical ventilation as weaning methods
Neurally adjusted ventilatory assist as an alternative to pressure support ventilation in difficult-to-wean patients: a single center randomized trial. Liu L. et al.	Compares two modalities of invasive mechanical ventilation as weaning methods
Neurally adjusted ventilatory assist versus pressure support ventilation: a randomized controlled feasibility trial performed in patients at risk of prolonged mechanical ventilation. Hadfield DJ. et al.	Compares two modalities of invasive mechanical ventilation as weaning methods
Effect of Pressure Support vs T-Piece Ventilation Strategies During Spontaneous Breathing Trials on Successful Extubation Among Patients Receiving Mechanical Ventilation: a Randomized Clinical Trial. Subirà C. et al.	Compares two modalities of invasive mechanical ventilation as weaning methods
Early Extubation for Patients With Acute Hypoxemic Respiratory Failure.	Registered trial
Characteristics of intensive care unit patients after planned extubation with Noninvasive ventilation and reintubation. Chang Y.	Not RCT, only abstract
