# Supplementary material for: Effects of early extubation followed by noninvasive ventilation versus standard extubation on the duration of invasive mechanical ventilation in hypoxemic non-hypercapnic patients: a systematic review and individual patient data meta-analysis of randomized controlled trials
Source: Crit Care. 2021 Jun 1;25:189. doi: 10.1186/s13054-021-03595-5 (PMC8169383; doi:10.1186/s13054-021-03595-5)
Supplement: Supplementary file 6 — Additional file 6. Criteria for readiness to wean and spontaneous breathing trails performed before randomization [file 13054_2021_3595_MOESM6_ESM.pdf]

Additional file 6. Criteria for readiness to wean and spontaneous breathing trails performed before randomization.

Study	Identification of readiness to wean	Ventilation before SBT	SBT before randomization	Criteria for SBT interruption	Number SBT failed before randomization
Ferrer et al. 2003	improvement or resolution of ARF<br><br>PaO_2 >60 mmHg at $FiO_2 \leq 0.4$ and PEEP ≤5 cmH_2O) receiving either assist-control or pressure-support ventilation<br><br>no fever (≥38º C) or hypothermia (<35º C) blood hemoglobin concentration ≥90 $g.L^{-1}$; no need for vasoactive drugs<br><br>normal consciousness (Glasgow coma score ≥13) with no need for sedative agents	assist-control or PSV	T-piece trail for 2 hours	RR > 35 breaths $minute^{-1}$<br><br>SpO_2 <90% (80% in chronic respiratory failure) at $FiO_2 \geq 0.4$<br><br>heart rate >140 or <50 beats $minute^{-1}$, or increases or decreases >20%<br><br>systolic blood pressure >180 or <70 mmHg, or increases or decreases >20%<br><br>decreased consciousness, agitation or diaphoresis<br><br>thoracic-abdominal paradoxical movement	Failed SBT for 3 consecutive days
Trevisan et al. 2008	improvement of the cause of ARF<br><br>correction of arterial hypoxemia PaO_2 > 60 mmHg and $FiO_2 \leq 0.4$<br><br>PEEP ≤ 5 cmH_2O in PSV<br><br>Psupp < 12cmH_2O<br><br>no requirement of vasoactive drugs<br><br>Glasgow coma scale ≥ 13<br><br>cough reflex<br><br>no need for sedation	PSV	T-piece trail for at least 30 min	SpO_2 < 90% (80% in chronic respiratory failure)<br><br>RR≥ 35 breaths $minute^{-1}$<br><br>HR > 140 or < 50 beats $minute^{-1}$ (or<br><br>increase or decrease of greater than 20%)<br><br>systolic arterial blood pressure > 180 mmHg or < 70 mmHg (or increase or decrease of greater than 20%)<br><br>rapid shallow breathing index > 105	1

Carron et al. 2014	Glasgow coma scale score ≥12<br>PaO_2/FiO_2 ≥ 150 mmHg<br>PEEP ≤ 5 cmH_2O<br>no vasopressor requirement<br>temperature <38°C<br>hemoglobin level ≥ 10 g dL^{-1}<br>no significant electrolytes alterations	PSV	Psupp 7cm H_2O for at least 30 min	agitation or impaired consciousness<br>RR> 35 breaths $minute^{-1}$ or an increase of 25% above baseline<br>hypoxemia with a decreased SaO_2<br>> 5% and/or PaO_2 less than 60 mm Hg with FiO_2 ≤ 0.35<br>$PaCO_2$ ≥ 60 mmHg<br>pH < 7.35<br>hemodynamic instability as demonstrated by a systolic arterial pressure less than 90 mmHg or greater than 180 mm Hg (or increase or reduction greater than 20% of baseline)<br>heart rate greater than 140 and less than 50<br>beats per minute (or increase or reduction greater than 20% of baseline), or the presence of arrhythmias not controllable with ordinary medical treatment.	1
Vaschetto et al. 2012	pressure support ventilation (PSV) with a total applied pressure ≤ 25 cmH_2O and a PEEP level between 8 and 13 cmH_2O<br>PaO_2/FiO_2 between 200 and 300 mmHg with a FiO_2 ≤ 0.6<br>$PaCO_2$ ≤ 50 mmHg and pH ≥7.35<br>respiratory rate ≤ 30/min	PSV	No SBT	/	/

	core temperature < 38.5°C<br><br>Glasgow coma scale = 11<br><br>cough on suctioning and need for tracheobronchial suctioning < 2 per hour				
Perkins et al. 2018	cooperative and pain free<br><br>good cough<br><br>PaO_2/FiO_2>24 kPa<br><br>PEEP <10 cmH_2O<br><br>Hb >7 g dL^{-1}, axillary temperature 36-38.5°C<br><br>vasoactive drugs reduced or unchanged over previous 24 h<br><br>spontaneous ventilatory frequency >6 min^{-1}	PSV according to patient comfort to achieve tidal volumes of 6-8 ml kg^{-1} ideal body weight and a respiratory rate < 30 breaths min^{-1}.<br><br>Once the patient is stable on PSV for at least 60 minutes, a SBT will be undertaken.	T-piece trial; CPAP or Psupp 5-7cm H_2O for at least 30 min	Signs of distress or fatigue according to International Consensus Conference on Weaning[a]	1
Vaschetto et al. 2019	pressure support ventilation (PSV) with a total applied pressure ≤ 25 cmH_2O and a PEEP level between 8 and 13 cmH_2O<br><br>PaO_2/FiO_2 between 200 and 300 mmHg with a $FiO_2 \leq 0.6$	PSV	No SBT	/	/

	tidal volume < 8 ml/kg of ideal body weight<br>$PaCO_2 \leq 50$ mmHg and pH ≥ 7.35<br>respiratory rate ≤ 30/min<br>core temperature < 38.5°C<br>Glasgow coma scale = 10T<br>cough on suctioning and need for tracheobronchial suctioning < 2 per hour				

ARF, Acute Respiratory Failure; CPAP, continuous positive airway pressure; FiO_2, inspired oxygen fraction; Hb, haemoglobin; HR, heart rate; $PaCO_2$, partial pressure of carbon oxide; PaO_2, partial pressure of oxygen; PEEP, positive end-expiratory pressure; Psupp, pressure support; PSV, pressure support ventilation; RR, respiratory rate; SpO_2, oxygen saturation; SBT, spontaneous breathing trial; Psupp, pressure support. [a] Boles JM, Bion J, Connors A, Herridge M, Marsh B, Melot C et al. Weaning from mechanical ventilation. Eur Respir J 2007;29(5):1033-1056.
