# Supplementary figures and images for: Effects of early extubation followed by noninvasive ventilation versus standard extubation on the duration of invasive mechanical ventilation in hypoxemic non-hypercapnic patients: a systematic review and individual patient data meta-analysis of randomized controlled trials
Source: Crit Care. 2021 Jun 1;25:189. doi: 10.1186/s13054-021-03595-5 (PMC8169383; doi:10.1186/s13054-021-03595-5)

**Additional file 7. Results of two-stage IPD-MA. Occurrence of reintubation (p value=0.83).**

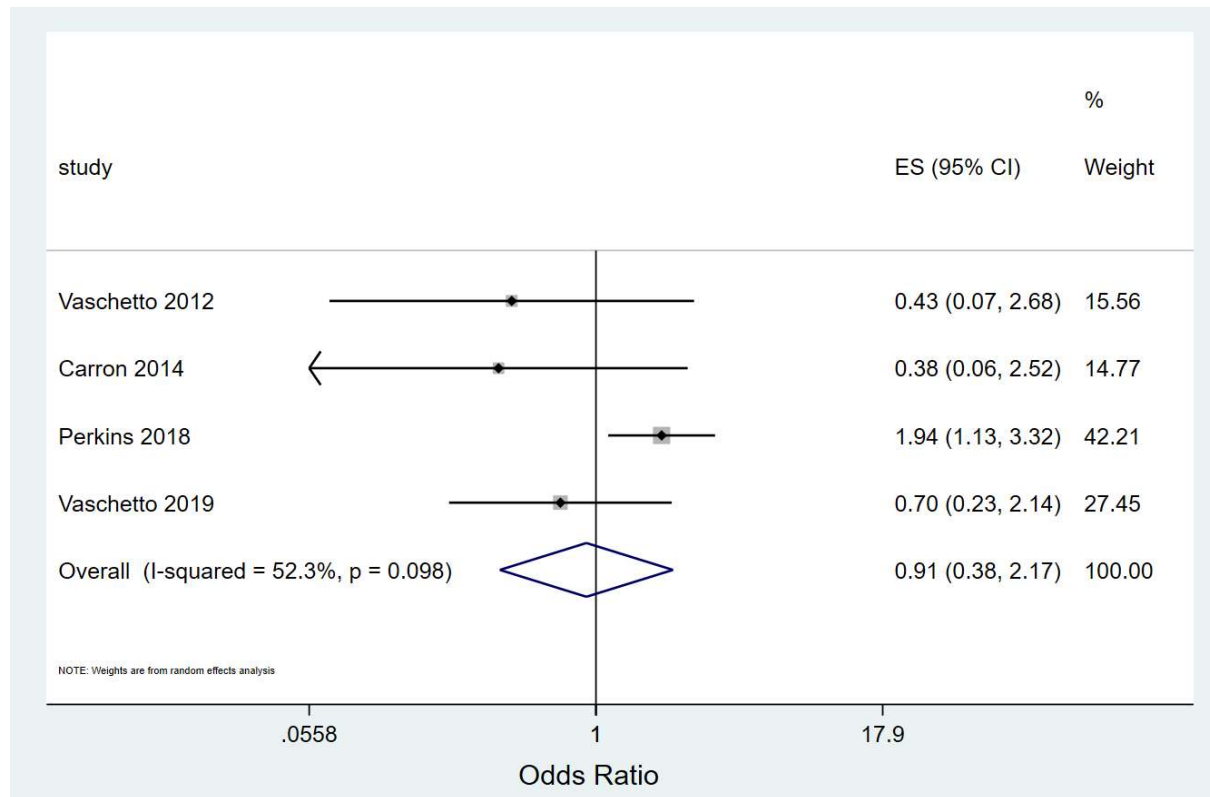

Supplement: Supplementary file 7 — Additional file 7. Results of two-stage IPD-MA. Occurrence of reintubation (p value=0.83) [file 13054_2021_3595_MOESM7_ESM.pdf]
