# Supplementary material for: Effects of early extubation followed by noninvasive ventilation versus standard extubation on the duration of invasive mechanical ventilation in hypoxemic non-hypercapnic patients: a systematic review and individual patient data meta-analysis of randomized controlled trials
Source: Crit Care. 2021 Jun 1;25:189. doi: 10.1186/s13054-021-03595-5 (PMC8169383; doi:10.1186/s13054-021-03595-5)
Supplement: Supplementary file 2 — Additional file 2. Letters sent to the authors [file 13054_2021_3595_MOESM2_ESM.pdf]

Additional file 2.

Dear Dr. ;

I am contacting you about a data sharing request for an individual patient data analysis.

We recently published in ICM a paper entitled “Early extubation followed by immediate noninvasive ventilation vs. standard extubation in hypoxemic patients. A Randomized Clinical Trial.” We now plan to conduct a pooled analysis of data from published studies to evaluate the effect of early extubation and noninvasive ventilation compared to standard weaning with the tube in place, on the duration of invasive ventilation and Intensive Care Unit length of stay in medical versus surgical hypoxemic non-hypercapnic patients.

Following a systematic literature search, we identified multiple relevant peer-reviewed studies including hypoxemic non-hypercapnic patients.

Your paper entitled ‘XY’ published in XY in 20XX was also identified by our research team. Therefore, I kindly ask you to share with us original data to enable an individual patient data analysis.

Would you be interested in joining this initiative? If yes, please reply this email and Dr. Rosanna Vaschetto shall contact you to discuss the variables needed and the transfer of the requested data.

All data will be handled according to applicable European legislation. We will not need all the dataset, but only some anonymous variables. The requested data will only be used for this manuscript.

From each participating research group, we propose adding one coauthor to the final manuscript, if appreciated.
